# Europe’s COVID-19 Crisis Response: A Race Well Run, But Not Yet Won

**DOI:** 10.1007/s10272-021-0981-x

**Published:** 2021-08-05

**Authors:** Alfred Kammer, Nathaniel Arnold

**Affiliations:** 1 European Department, International Monetary Fund, 700 19th Street, N.W., 20431 Washington, DC, USA; 2 Headquarter 2, 1900 Pennsylvania Ave NW, 20431 Washington, DC, USA

## Abstract

While Europe’s response to the pandemic has been laudable, there remains more to be done in order to prevent economic scarring and ensure a robust recovery.

## A laudable response to the pandemic

The COVD-19 crisis has been the biggest global economic shock since World War II. Unlike the "global" financial crisis – which was really a US and European financial crisis that spilled over to the rest of the world – the pandemic was truly an exogenous global shock.

Many countries reacted quickly with mobility restrictions and lockdowns as the pandemic swept across the globe in early 2020. These initial containment measures dealt a blow to the incomes of businesses and workers across the economy, though the intensity of the impact differed across sectors – with tourism and hospitality clearly suffering more and being less able to adapt. Moreover, the shock was severe but expected to be temporary due to the success of activity restrictions on reducing new COVID-19 cases in the first wave of the pandemic, as well as rapid development of vaccine candidates.

Governments, especially in advanced economies, countered the COVID-19 crisis with unprecedented levels of policy support. In Europe, policies aimed at preventing the unnecessary destruction of businesses and jobs and maintaining the structure of the economy in the face of a temporary exogenous shock. And the policy response so far – at both the national and European levels – has been incredibly successful.

The monetary and financial regulatory policy response helped prevent financial markets from seizing up as the pandemic struck. It kept credit – the lifeblood of the economy – flowing, with lending growth to firms jumping from 2.5% in February 2020 to over 6.5% in May in the euro area.

**Alfred Kammer**, International Monetary Fund, Washington DC, USA.

**Nathaniel Arnold**, International Monetary Fund, Washington DC, USA.

At the same time, fiscal support for workers and businesses helped prevent a massive rise in unemployment and a cascade of bankruptcies. This has – to a large extent – preserved the fabric and structure of the economy in the face of massive uncertainty. These policies helped keep household disposable income in the euro area from declining between 2019 and 2020, despite a 6.6% drop in real GDP. The public sector stepped up to shoulder the burden of the shock and tried to make households and businesses whole – a task made easier by the strength of the existing social safety nets in Europe, which were able to be quickly expanded where needed to meet the challenges of the COVID-19 crisis.

For instance, job retention schemes were a critical part of the response. These schemes pay part of an employee's normal wages if a business keeps them employed but reduces their hours. In this way, businesses can temporarily reduce labour costs in the face of weaker demand without laying off workers. At the same time, workers' incomes are maintained even if the working hours are reduced. Moreover, by maintaining the worker-job bond, job-specific human capital is preserved and long-lasting economic scarring due to the loss of human capital is reduced.

Many EU countries had job retention schemes in place already and expanded them during the crisis to cover a broader range of workers (e.g. gig workers), while others were able to quickly add such programmes to their social safety nets. On average, EU countries are estimated to have spent nearly 2% of GDP on job retention schemes in 2020. The benefits of such schemes are readily apparent from the unemployment numbers. While hours worked in the second quarter of 2020 fell more than 15% below late-2019 levels in the EU, the employment rate fell less than two percentage points and the unemployment rate ticked up one percentage point. These job retention schemes were supported at the EU level with €100 billion in low-cost loans to countries.

In contrast, the US, which did not have job retention schemes in place, saw a sharp spike in the unemployment rate in the second quarter of 2020, going from 4.4% to 14.8% between March and April, before steadily declining. As a result, the annual average unemployment rate in the US more than doubled in 2020, necessitating higher spending on unemployment benefits, which was similar to the average amount spent in EU countries on job retention schemes.

European countries also announced an array of measures to support businesses, including “above-the-line” measures like tax cuts, as well as “below-the-line” measures like equity support, loans and loan guarantees. For the latter, euro area countries announced programmes worth 17% of GDP in 2020. Even though the eventual take up of these programmes only amounted to about 5% of GDP, these measures played an important role in shoring up confidence and convincing banks, which entered the crisis in a strong position, to continue lending in the face of a sharp drop in economic activity.

Despite the large fiscal response, Europe on average suffered a greater drop in real GDP than the US did. While comparisons between fiscal responses in the US and the EU are not straightforward, taking into account the more robust social safety nets in EU countries and “below-the-line” measures, they provided comparable degrees of fiscal support in 2020. The pandemic had a larger growth impact on many European countries, but this was mainly due to factors besides the fiscal response. These factors include different initial underlying growth rates, the structure of the economy – with many of the hardest hit European countries more dependent on sectors most impacted by the pandemic like tourism – the severity of activity restrictions and the ability to adapt to the pandemic, such as through teleworking.

## A robust transition to the post-pandemic world

Now, with vaccine rollouts accelerating, the prospects for a return to something approximating normalcy look good. But the economy will not just snap back to its prepandemic form. There will be shifts in the structure of the economy, partly driven by deeper technological and environmental changes. Moreover, achieving a greener, more digital and inclusive economy will require transformations in many sectors. Policies must help facilitate these transformations.

Labour and product markets in the EU are generally more rigid than those in the US, and European firms lack access to venture capital and other forms of financing that many US firms enjoy. These shortcomings will inhibit the capacity of European economies to respond to the shifts in the economic landscape occasioned by the pandemic and coming economic transformations.

Less flexible labour markets can hinder the flow of workers from declining firms and sectors to new and expanding ones, serving as a drag on growth. Moreover, if government support for firms is withdrawn too quickly, it could lead to the insolvency of potentially viable firms, while if continued support takes the form of debt, it may leave many viable firms suffering from a debt overhang, limiting their capacity to invest and expand as demand picks up. This could weigh down the recovery and result in very costly output losses relative to the pre-pandemic trajectory of the economy.

Thus, in the second phase of the crisis response, as lockdowns end, spending on generalised support that is demand driven, such as job retention schemes, will automatically decline. In this second phase, there is a greater need for supply-side oriented spending to accelerate the recovery. This argues for replacing some of the automatic decline in spending on lifelines with spending aimed at facilitating the flow of labour and capital out of declining sectors and into expanding and new ones.

Higher spending on things like training for displaced workers, alongside incentives for hiring and investment in expanding or new sectors, can do a lot to facilitate the necessary flows and minimise the long-term costs of the pandemic. This should be supplemented with targeted transfers to vulnerable households that are more likely to suffer income losses as emergency lifelines are wound down. This would also help mitigate the rise in inequality caused by the disproportionately larger impact of the pandemic on more vulnerable households.

With regard to the corporate sector, there is also a need for more equity support for viable firms. The IMF estimated a solvency gap for European enterprises of between 2% and 3% of GDP.

In the Spring 2021 Regional Economic Outlook Update for Europe, IMF staff calculated that a package of measures along the lines described above over 2021-22 could boost GDP in 2022 by two percentage points and cut medium-term output losses relative to the pre-pandemic trend in Europe by more than half.

Since then, a number of countries have introduced further fiscal support packages. While these packages have been mainly geared towards extending lifelines in the face of renewed lockdowns, they also contain elements that will support the reallocation of factors. For example:

- part of Germany’s supplemental budget of 1.7% of GDP expanded subsidies for apprenticeships and increased grants allocated to firms, as well as provided a one-off boost to basic income support;
- in Italy, a significant chunk of the 2% of GDP fiscal package approved recently will provide grants to businesses and additional one-off support for workers in hard-hit industries like tourism.

These measures are welcome, but as the recovery gets underway, they should be complemented by further efforts to help workers transition to new careers. We must also ensure that young and low-skilled workers – whose employment prospects were some of the hardest hit by the crisis – are not left behind. In this respect, France's recovery plan provides some good examples of such policies. It offers subsidised training to workers in job retention schemes and expands funding for training targeted at low-skilled youth. In addition, it introduces a programme of hiring subsidies for younger workers to help get them into the labour market.

Of course, fiscal policy alone is not the answer.

The Recovery and Resilience Facility (RRF) in the Next Generation EU (NGEU) recovery package is an important element in the third phase of the response to the pandemic, aimed at strengthening the recovery, promoting convergence between countries and helping them transform their economies. Grants and loans from the RRF will support EU countries investments in critical areas, like climate change and digitalisation. Higher spending financed by the RRF should be a catalyst and not a substitute for structural reforms. As it will take time for these reforms to bear fruit, it is important to start making progress on them now.

If well spent, simulations suggest that RRF-financed expenditure could boost EU output by up to 1.5 percentage points by 2023, relative to a scenario without the RRF. While some of this is already incorporated into forecasts by institutions like the IMF and European Commission, there is a potential for growth to surprise to the upside if investments are effectively implemented and accompanied by robust structural reforms.

## Reducing vulnerabilities and strengthening the EU fiscal framework

One cannot ignore the impact that the crisis has had on government debt levels, especially in those countries that entered the pandemic with already elevated debt ratios. Once a robust economic expansion is firmly in place, high-debt countries will need to embark on a path of gradual, but steady, fiscal adjustment. This will help restore fiscal buffers to respond to future shocks. In many high-debt countries, the growth impact of a gradual adjustment will be mitigated over the next few years as RRFs grants bolster spending. Making the composition of fiscal policy greener, more growth-friendly and inclusive would also help.

The impact of the crisis on government debt also has implications for the EU fiscal rules. The current framework suffers from excessive complexity, poor transparency and weak compliance. Moreover, once the general escape clause in the rules is deactivated, a strict application of the current rules would require unfeasibly large fiscal tightening in high-debt countries. Hence, now, while the escape clause is activated, would be a good time to reform the rules.

The IMF is currently taking a fresh look at how the rules should be reformed in light of the pandemic. Some of the principles that should guide reform efforts include: First, a reform should aim to simplify the rules to make them easier to understand, communicate, monitor and enforce. Second, the fiscal rules need to balance debt sustainability and economic stabiliastion objectives. They must help build better buffers in good times but cannot be a straitjacket of procyclical tightening in recessions. Finally, in the longer run, incentives for compliance with the rules could be strengthened by developing a permanent central fiscal stabilisation capacity.

## Conclusion

While Europe's response to the pandemic has been laudable, there remains more to be done in order to prevent economic scarring and ensure a robust recovery. The early part of the recovery that we are entering now is a critical period. Greater focus is needed on efforts to facilitate the reallocation of labour and capital from declining firms and sectors to new and expanding ones as the recovery gets underway. EU recovery funds can complement such measures with more medium-term support for investments to support an economic transformation and accelerate the green and digital transitions. Moreover, EU funds can help mitigate the growth impact over the next few years of the gradual, but steady, fiscal adjustments that will be needed in high-debt countries. Finally, the EU fiscal rules should be reformed while the general escape clause is in place, to ensure that they are fit for purpose in the post-pandemic world.
